# A Bayesian QTL linkage analysis of the common dataset from the 12^th ^QTLMAS workshop

**DOI:** 10.1186/1753-6561-3-s1-s4

**Published:** 2009-02-23

**Authors:** Marco CAM Bink, Fred A van Eeuwijk

**Affiliations:** 1 Biometris, Wageningen University & Research centre, Bornsesteeg 47, 6708 PD, Wageningen, Netherlands; 2 Centre for BioSystems Genomics, PO Box 98, 6700 AB Wageningen, Netherlands

## Abstract

**Background:**

To compare the power of various QTL mapping methodologies, a dataset was simulated within the framework of 12^th ^QTLMAS workshop. A total of 5865 diploid individuals was simulated, spanning seven generations, with known pedigree. Individuals were genotyped for 6000 SNPs across six chromosomes. We present an illustration of a Bayesian QTL linkage analysis, as implemented in the special purpose software FlexQTL. Most importantly, we treated the number of bi-allelic QTL as a random variable and used Bayes Factors to infer plausible QTL models. We investigated the power of our analysis in relation to the number of phenotyped individuals and SNPs.

**Results:**

We report clear posterior evidence for 12 QTL that jointly explained 30% of the phenotypic variance, which was very close to the total of included simulation effects, when using all phenotypes and a set of 600 SNPs. Decreasing the number of phenotyped individuals from 4665 to 1665 and/or the number of SNPs in the analysis from 600 to 120 dramatically reduced the power to identify and locate QTL. Posterior estimates of genome-wide breeding values for a small set of individuals were given.

**Conclusion:**

We presented a successful Bayesian linkage analysis of a simulated dataset with a pedigree spanning several generations. Our analysis identified all regions that contained QTL with effects explaining more than one percent of the phenotypic variance. We showed how the results of a Bayesian QTL mapping can be used in genomic prediction.

## Background

The 12^th ^QTLMAS workshop included a section that focussed on discussions about analyses of a simulated data set. The common dataset [1] comprised a total of 5865 diploid individuals, spanning seven generations, with known pedigree. Only the first four generations, containing 4665 individuals, were phenotyped for a single trait. In the founder population, 15 males and 150 females were present (Table 1). In the subsequent generations, numbers of males and females were comparable. For genotyping, 6000 SNPs across six chromosomes were scored. The dataset was simulated to allow the first four generations to be used for QTL detection (by association, linkage or combinations thereof). No phenotype was given for the last three generations since these were included for genomic selection purposes. The objective of our contribution is to present the results of a Bayesian analysis fitting multiple QTL simultaneously by exploiting linkage information.

**Table 1:** Numbers of individuals and means of trait phenotypes across generations of the simulated dataset.

**Generation**	**pedigree**			**phenotypic mean**		
	**male**	**female**	**total**	**male**	**female**	**average**
0	15	150		2.18	0.89	1.01
1	770	730		1.39	1.55	1.47
						
			1665			
2	762	738		1.25	1.42	1.33
3	717	783		1.38	1.26	1.32
						
			3000			
			4665			
4	162	238		n.a.	n.a.	
5	156	244		n.a.	n.a.	
6	196	204		n.a.	n.a.	
						
			1200			

	2778	3087	5865	1.34	1.37	1.36

## Methods

### Phenotypic data

The quantitative trait was measured on 4665 individuals with mean and variance estimated to be 1.36 and 4.42, respectively (Table 1). The generation number and sex of each individual were provided as non-genetic variables that might be included in the analyses. Individuals in generations 4–6 did not have phenotypes available and these individuals were excluded from the linkage analyses. Preliminary analyses revealed that across all generations jointly there was no sex effect on the phenotype, however, in the oldest generation (0) the phenotypic means of males and females differed, i.e., 2.18 versus 0.89 (Table 1). The phenotypic means for generations 0 and 1 were relatively low (1.01) and high (1.47), respectively.

### Marker data

The haplotype data on the 165 individuals of generation 0 were analysed by HapBlock software [2] to identify putative haplotype blocks. Neither this combined analysis of males and females jointly nor the analyses of males (n = 15) and females (n = 150) separately revealed clear Linkage Disequilibrium structures to exist across the genome and therefore a pragmatic thinning of markers was applied. Two subsets from the total of 6000 SNP markers were selected by picking every 10^th ^or 50^th ^SNP along the genome, resulting in 600 or 120 loci, respectively.

### Statistical model for linkage analysis

The QTL was assumed to be bi-allelic, allowing three genotypes to be distinguished, i.e., QQ, Qq, and qq, having genotypic values equal to + *α*, *δ *and -*α*, respectively. The variables *α *and *δ *represent the additive and dominance effects of a single gene. The allele frequency of the positive allele Q is denoted by *f*_*α*_, and may take any value between 0 and 1 with equal prior probability.

The linear model in our Bayesian analysis is similar to Bink et al. [3] and may be given as follows,

(1) $$y\sim N\left(X\beta +W\alpha_{qtl},\sigma_{e}^{2}\right)$$

where *β *is a vector containing an overall mean (*μ*) and all non-genetic variables affecting the trait of interest, i.e., sex and generation. The vectors *α*_*qtl *_represent the additive and dominant genetic contributions of a QTL. The incidence matrices **X**, **W **connect the phenotypes to non-genetic and QTL variables, respectively. The entry values of matrix **W **depend on the genotype assigned to each individual. For the genotypes {QQ, Qq, qq} these values equal {+1, 0, -1} and {0,1,0}, for additive and dominant effects, respectively. Note that the sign of QTL effects are relative to the QTL genotypes and therefore a QTL cannot be assigned to contribute positively or negatively to the trait. The number of columns in **W **depends on the number of QTL in the model. Treating the number of QTL as a random variable in a Bayesian framework was facilitated by the use of the Reversible Jump sampler [4,5]. The positions of putative QTL are specified in centiMorgan (cM) [6] and denoted by *λ*_*QTL*_.

The prior distributions on model parameters were taken similar to those by Bink et al. [3], here we only report results for the prior assumption that the expected number of QTL, i.e., the mean of the Poisson distribution, equals five. The influence of the prior mean appeared to be minimal when model selection was based on Bayes Factors for competing models with different numbers of QTL (results not shown).

### Joint posterior distribution

Let **P **and **M **denote the pedigree and marker data, respectively, and $\theta =\left(\beta ,\alpha_{QTL},\sigma_{e}^{2}\right)$, then the joint posterior distribution of all unknowns can be written as (omitting matrix **X**),

(2) $$\begin{array}{c} p\left(\theta ,f_{\alpha },N_{QTL},\lambda_{QTL},W|y,M,P\right)\\ \propto p\left(y|\theta ,W\right)p\left(W|f_{\partial },N_{QTL},\lambda_{QTL},M,P\right)p\left(\theta ,f_{\alpha },N_{QTL},\lambda_{QTL}\right) \end{array},$$

where the first term on the right hand side is the conditional distribution of the phenotypic data given all unknowns from (1). The second term is the probability distribution of QTL genotypic states (genotypes) conditional on the number and locations of QTL, the QTL allele frequencies, and the pedigree and marker data. The final term in equation (2) is the joint prior distribution of the model variables.

### Posterior computations

We used the FlexQTL™ software  that performs Markov chain Monte Carlo (MCMC) simulation [7-9] to obtain draws from the joint posterior distribution. For all simulations, a Markov chain was executed for 500 K iterations and every 100^th ^iteration samples were stored for posterior inference. The chromosomes were divided into small intervals (1 cM-bins) and the number of QTL per bin per cycle was used to calculate the posterior QTL intensity [10]. This procedure was used independent from the marker density (1 or 5 cM spacing). For the posterior inference on the chromosomal positions of the QTL we use 0.90 Highest Posterior Intensity (abbreviated to HPI90) [3]. Posterior mean and 90% quantiles for QTL effects were computed for those chromosomal bins that contained sufficient intensity (samples).

The samples of QTL genotypes of the first 30 individuals of the dataset, i.e., 15 males and the first 15 females of generation 0, were stored and used to compute posterior probabilities along the genome using 5 cM bins. A color-coding was applied to indicate probability of genotype assignment,

(3) $$\{\begin{array}{lll} P\left(QQ|y\right) & >0.8(0.6) & dark(light)\;red\\ P\left(Qq|y\right) & >0.8(0.6) & dark(light)\;green\\ P\left(qq|y\right) & >0.8(0.6) & dark(light)\;blue\\ else & & gray \end{array}$$

The individuals' genotypes and QTL effects (additive and, if included, dominance) were multiplied to estimate the individuals' genotypic values (or breeding value) along the genome. These breeding values were subsequently weighted by the posterior evidence of a QTL being present at a specific chromosomal bin. A heat-coloring scheme was applied where the degree of redness (blueness) indicated more positive (negative) values. The additive and dominant genetic variance explained by all QTL jointly were calculated as

(4) $$\sum_{j}^{N_{QTL}}2\left(f_{\alpha }(1-f_{\alpha }){\left[\alpha_{j}+\delta_{j}(1-2f_{\alpha })\right]}^{2}\right),$$

(5) $$\sum_{j}^{N_{QTL}}\left({\left[2f_{\alpha }(1-f_{\alpha })\delta_{j}\right]}^{2}\right),$$

where Hardy Weinberg equilibrium was assumed in the initial founder population [11] and linkage equilibrium among QTL.

### Model selection

In respect of model selection, we use Bayes factors [12] as a measure of evidence coming from the data for different QTL models. More specifically twice the natural logarithm (2ln) of a Bayes Factor was used as this was on the same scale as the familiar deviance and likelihood ratio test statistics. The Bayes factor is the ratio of the marginal likelihood under one model to the marginal likelihood under a second model and was computed from the prior and posterior odds ratios for the competing models[12]. The Bayes factors for two competing models can be interpreted as follows: 2ln(BF) = [0–2, 2–5, 5–10, >10] corresponds to [hardly any, positive, strong, decisive] evidence against 1^st ^model, respectively. QTL with positive or stronger evidence are reported in this study.

### Types of genetic models

The default in this study was the additive genetic model with a prior mean for the number of QTL equal to 5, denoted as **Q5a**. This prior mean reflects our expectation that there are likely 5 QTL affecting the quantitative trait in an additive manner. The models in which the QTL affect the trait in both additive and dominant manner are denoted **Q5ad**. As outlined above, we studied two marker densities, i.e., 1 cM and 5 cM, and we explored the power to map QTL when only part of the phenotypic data was used, i.e., only data on the first 2 generations of individuals.

## Results

### Estimates of heritability

The estimates of heritability from QTL models were 0.3 or somewhat higher when all data was used and just below 0.3 when only the first 2 generations of individuals were considered (Table 2). A similar estimated heritability was obtained when fitting a purely polygenic model (ignoring marker data) to the phenotypic and pedigree data (results not shown). Fitting a model with both multiple QTL and a random polygenic effect simultaneously resulted in an estimated polygenic variance near zero, which indicates that the QTL account for all additive genetic variance underlying the phenotypic trait.

**Table 2:** Posterior inference on genetic parameters from several QTL models

	nPHE	mPHE	vPHE	vERR	nQTL	vQTL	H2
01 cM_Q5a	4665	1.36	4.42	3.03	13.6	1.50	**0.33**
01 cM_Q5ad	4665	1.36	4.42	3.03	13.6	1.52	**0.33**
							
05 cM_Q5a	4665	1.36	4.42	3.06	12.8	1.43	**0.32**
05 cM_Q5ad	4665	1.36	4.42	3.01	13.5	1.53	**0.34**
							
01 cM_Q5a_2G	1665	1.42	4.46	3.29	8.8	1.33	**0.29**
05 cM_Q5a_2G	1665	1.42	4.46	3.33	8.3	1.26	**0.27**

01 cM/05 cM = marker distance; a/ad = QTL with additive or additive & dominant effects; 2 G = only 1^st ^two generations of individuals included.

nPHE = number of phenotypes; mPHE = mean of phenotypes; vPHE = variance of phenotypes; vERR = posterior mean of error variance; nQTL = posterior mean of number of QTL; vQTL = posterior mean of QTL variance; and H2 = posterior mean of heritability.

### Number of QTL

The posterior mean estimates for the number of QTL varied from 12.8 to 13.6 when using all data and an estimate of 8.6 when using only the 1^st ^two generations (Table 2). The number of QTL per chromosome with clear positive evidence varied from 0 on chromosome 3 and 6 up to 4 on chromosome 4 when using the Bayes Factor estimates for the most likely QTL model (Table 3). The evidence for QTL on chromosome 3 was only present when analysing all phenotypes with a lower marker density, i.e., in models 5 cM_Q5a and 5 cM_Q5ad. The 5 cM density map resulted in less QTL identified, especially on chromosome 1, 2, and 4 (Table 3). The use of only 2 generations of phenotypes resulted in lowest numbers of QTL identified, i.e., only 3 with strong evidence.

**Table 3:** Estimates of Bayes Factors of QTL models (favouring model M1 over model M0) per chromosome (chr)

	**chr 1**			**chr 2**			**chr 3**	**chr 4**			**chr 5**		
***M0***	**0**	**1**	**2**	**0**	**1**	**2**	**0**	**0**	**1**	**2**	**3**	**0**	**1**
***M1***	**1**	**2**	**3**	**1**	**2**	**3**	**1**	**1**	**2**	**3**	**4**	**1**	**2**
**01 cM_Q5a**	na	27	3	na	13	3	na	na	na	24	8	26	3
**01 cM_Q5ad**	na	9	3	na	12	3	na	na	na	10	5	25	3
**05 cM_Q5a**	na	9	4	na	12	na	4	na	27	4	na	11	4
**05 cM_Q5ad**	19	8	4	21	7	na	4	na	24	5	4	9	na
**01 cM_Q5a_2G**	26	na	na	26	na	na	na	7	3	na	na	25	3
**05 cM_Q5a_2G**	11	na	na	9	na	na	na	4	na	na	na	7	^na^

01 cM/05 cM = marker distance; a/ad = QTL with additive or additive & dominant effects; 2 G = only 1^st ^two generations of individuals included.

na = not available, i.e., the models M0 and/or M1 were insufficiently sampled for posterior inference.

### Positions of QTL

**Figure 1:**
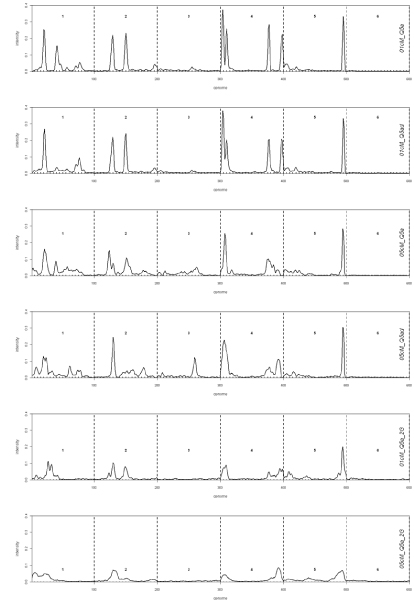
**Estimated posterior intensity of QTL positions along the genome (6 chromosomes, each of length 100 cM) for the QTL models of Table 2**.

The estimated intensity profile of indicated QTL had narrow peaks when all phenotypic data and a 1 cM marker density was used (Figure 1). For this marker density, the estimated position of the 2^nd ^QTL on chromosome 1 was bimodal and 2 closely linked QTL were identified at the start of chromosome 4. The marker density of 5 cM resulted in much less narrow and lower QTL intensity profiles, while using phenotypic data partially (1665 records – Table 1) resulted in rather flat profiles (Figure 1).

For the model 1 cM_Q5a, the length of the twelve QTL regions with strong evidence varied from 4 up to 22 cM (Table 4). The boundaries of these regions were based upon the Highest Posterior Intensity inference, i.e., all values within these regions were never lower than values outside these regions. Note that the number of regions was based upon the Bayes Factors (Table 3). The intensity of some regions were greater than 1.0 as these regions sometimes harbored more than 1 QTL at the same time.

**Table 4:** Estimates for QTL locations and contributions for model 1 cM_Q5a

ID	Linkage Group	Start Length		Mode	Intensity	additive effect	variance	weighted variance
1	1	9	14	21	1.14	0.55	0.14	0.16
2	1	38	10	41	0.92	0.67	0.09	0.08
3	1	68	16	76	0.52	0.30	0.05	0.02
4	2	24	9	29	1.06	0.58	0.16	0.17
5	2	44	11	50	1.08	0.46	0.10	0.11
6	2	91	8	99	0.23	0.31	0.05	0.01
7	4	1	4	4	1.21	0.78	0.30	0.37
8	4	5	19	10	1.19	0.55	0.15	0.18
9	4	73	6	77	1.04	0.50	0.12	0.13
10	4	93	6	98	0.92	0.41	0.09	0.08
11	5	1	22	2	0.60	0.35	0.06	0.03
12	5	93	3	95	1.00	0.72	0.24	0.24

Weighted variance = variance weighted by intensity

### QTL effects and variance

**Figure 2:**
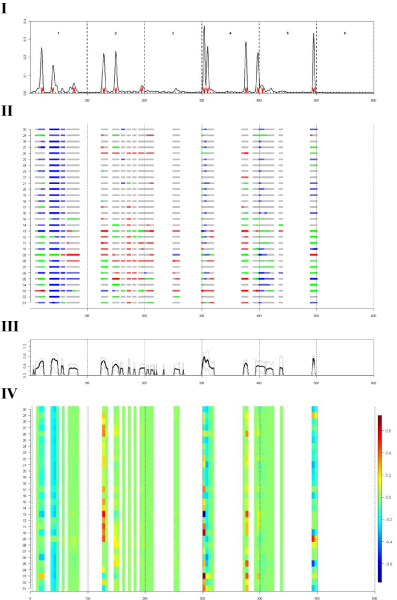
**Posterior inference on QTL characteristics along the genome for the model 1 cM_Q5a**. (I) Posterior QTL intensity; (II) Posterior genotype probabilities of 1^st ^thirty individuals of the dataset (*QQ = red; Qq/qQ = green; qq = blue; ambiguous = gray*, see also equation (3)); (III) Estimates of posterior mean (black line) and 90%quantiles (gray lines) of additive QTL effects; (IV) Estimated breeding values of 1^st ^thirty individuals.

The posterior mean estimates of additive QTL effects in the twelve regions varied from 0.31 up to 0.78 (Table 4). The posterior 90% quantiles (of the distribution within bins) for the additive QTL effects are depicted in Figure 2 and the QTL at the end of chromosome 5 had the tightest quantile region.

### QTL genotypes and breeding values

The posterior probabilities of the first 30 individuals along the genome are depicted for bins with increased posterior QTL intensity (Figure 2). Assignment of individuals' QTL genotypes for regions with high QTL intensity was often possible, e.g., first 2 QTL on chromosome 1 and last QTL on chromosome 5. However, assignment was poor for other QTL regions, e.g., QTL on chromosomes 2 and 4.

The colour-representation of the estimated breeding values showed only a limited number of regions with clear variation in breeding values estimates (Figure 2). The QTL at the start of chromosome 4 caused the largest variation in breeding values, which was consistent with the amount of variance explained by the QTL (Table 4).

## Discussion

The genetic models studied assumed either QTL acting additively or additively and dominantly. Allowing dominance did not result in a different number of QTL identified nor did the locations of the QTL change dramatically. For the 1 cM scenarios the main difference were the QTL intensity profiles on chromosome 1 (Figure 1), i.e., the model allowing dominance revealed more evidence for a QTL in the 2^nd ^half of the chromosome. Also, the estimates of dominance effects were close to zero for almost all QTL (results not shown). The inclusion of epistatic interactions in our Bayesian QTL framework is in progress.

The comparison to the simulated QTL positions (provided after the workshop) revealed that our Bayesian analyses correctly identified almost all QTL that explained more than 1% of the phenotypic variance [13]. The QTL simulated at 74 cM-chr2, 60 cM-chr3, and 36 cM-chr4 were not reported in our study. The QTL at 74 cM-chr2 had a rather low minor allele frequency (0.16) in the population [1] and that could have been the reason that this QTL was missed in our analyses. For the QTL at 60 cM-chr3 there was increased, but not convincing, posterior evidence (Figure 2). The QTL at chr4 was missed although we reported another QTL positioned closer to the start of chromosome, i.e., at 10 cM. The simulated QTL jointly explained 30% of the phenotypic variance and this value corresponds well with the heritability estimates from our analyses (Table 2).

The rapidly growing availability of SNP markers introduces new types of datasets that can be analysed to find associations between genotype and phenotype. Instead of a limiting factor, the number of markers is now overloading the statistical methods for QTL mapping. We thinned the number of available SNP markers down to a number that could be more easily handled in our Bayesian linkage analyses. This thinning was ad-hoc as a survey on haplotype patterns among generation 0 individuals did not reveal large Linkage Disequilibrium stretches. Reducing the resolution of SNP markers down to 5 cM introduced a severe loss of power to identify and map QTL (Table 3, Figure 1). The marker haplotype data provided complete information on linkage phase among subsequent markers which is not yet utilized in the current FlexQTL software.

An important research item of the simulated data set was to predict the breeding values for non-phenotyped juvenile individuals. Here, we did not include these individuals as inclusion would increase computation time but not increase the power of QTL mapping. The FlexQTL software allows the storage of genotype samples on all individuals and thereby allows genomic prediction for juveniles, but computation and storage capacity may become limited and we plan to extend the software on this issue.

## Conclusion

We successfully identified 12 chromosomal regions with substantial evidence for harbouring QTL affecting the quantitative trait of interest. These QTL explained 30 percent of the total phenotypic variance. Our Bayesian approach produces posterior individuals' QTL genotype probabilities and by fully accounting for posterior uncertainty in presence and size of QTL also predicts genome-wide breeding values.

## Competing interests

The authors declare that they have no competing interests.

## Authors' contributions

MB performed linkage analyses. MB and FE wrote and discussed the manuscript.
